# Delayed sleep-wake rhythm is associated with cognitive dysfunction, social dysfunction, and deteriorated quality of life in patients with major depressive disorder

**DOI:** 10.3389/fpsyt.2022.1022144

**Published:** 2022-11-07

**Authors:** Yoshikazu Takaesu, Yuta Kanda, Yu Nagahama, Ayano Shiroma, Miho Ishii, Tasuku Hashimoto, Koichiro Watanabe

**Affiliations:** ^1^Department of Neuropsychiatry, Graduate School of Medicine, University of the Ryukyus, Nishihara, Japan; ^2^Department of Neuropsychiatry, Kyorin University School of Medicine, Tokyo, Japan; ^3^Department of Psychiatry, School of Medicine, International University of Health and Welfare, Narita, Japan; ^4^Department of Psychiatry, Sodegaura Satsukidai Hospital, Sodegaura, Japan; ^5^Department of Psychiatry, Graduate School of Medicine, Chiba University, Chiba, Japan

**Keywords:** major depressive disorder, circadian rhythm sleep-wake disorders, delayed sleep-wake rhythm, cognitive dysfunction, social dysfunction, quality of life

## Abstract

**Background:**

The delayed sleep-wake phase is commonly observed in major depressive disorder (MDD) and thought to be associated with functional impairments. This study aimed to evaluate the relationship between the delayed sleep-wake phase, cognitive dysfunction, social dysfunction, and quality of life in patients with MDD.

**Methods:**

This cross-sectional design included 33 outpatients with MDD. Objective sleep-wake rhythm was assessed by actigraphy. Functional impairments were evaluated by the Japanese version of the Brief Assessment of Cognition in Schizophrenia (BACS-J), World Health Organization Disability Assessment Schedule (WHO-DAS), and Euro QOL 5 dimensions (EQ5D).

**Results:**

Actigraphic assessment of the delayed sleep-wake phase (midpoint of sleep) was significantly correlated with the composite score of the BACS-J (*r* = –0.489, *p* = 0.010), WHO-DAS score (*r* = 0.466, *p* = 0.014), and EQ5D score (*r* = 0.472, *p* = 0.013). No significant correlation was found between the other actigraphic sleep parameters (sleep latency, total sleep time, and sleep efficiency) and functional impairments.

**Conclusion:**

Our study’s results suggested that the delayed sleep-wake phase is associated with cognitive dysfunction, social dysfunction, and deteriorated quality of life in patients with MDD. Clinicians should pay attention to the sleep-wake rhythm in patients with MDD in clinical settings.

## Introduction

Sleep disturbances are common in major depressive disorder (MDD). More than 90% of patients with MDD have sleep disturbances (1). Previous polysomnography studies in MDD patients have observed shortened REM sleep latency, increased REM density, and decreased slow wave sleep (2). Sleep disturbances were reported to be associated with the severity of depressive symptoms (3) as well as increasing the risk of suicide (4) in patients with MDD. In addition, a longitudinal study suggested that the residual sleep disturbances after remission of depressive symptoms could be increasing the risk of relapses and recurrences (5). Insomnia and circadian rhythm dysfunction, especially delayed sleep-wake rhythm, were commonly observed in patients with MDD (6). Recent studies have suggested that the delayed sleep-wake phase is likely to worsen depressive symptoms and cognitive dysfunctions (7). However, few studies have assessed influences of the delayed sleep-wake phase on depressive symptoms and functional impairment in patients with MDD.

Cognitive dysfunction is a common functional impairment of various psychiatric disorders, including schizophrenia (8), bipolar disorder, and MDD (9). Many studies have shown that cognitive dysfunction is associated with occupational and social functional impairments in patients with MDD (10–12). In the treatment course of MDD, cognitive dysfunction can improve with adequate treatment but may also partially remain even after remission of depressive symptoms (13–15). Notably, a longitudinal study reported that residual cognitive dysfunction in remitted patients with MDD might be a predictor for recurrence of the depressive symptoms (16). Thus, clinicians must pay attention to residual cognitive dysfunction in clinical settings.

Few studies have suggested the relationship between sleep disturbances and cognitive dysfunction in patients with MDD (6). However, there could be a possible pathophysiological relationship between sleep disturbances and cognitive dysfunction. Therefore, this study aimed to investigate the relationship between sleep disturbances and cognitive dysfunction in patients with MDD.

## Materials and methods

### Ethics statement

This study was conducted in accordance with the Declaration of Helsinki and approved by the ethics committee of Kyorin University. Written informed consent was obtained from the patients.

### Participants

Patients with MDD were diagnosed by fully trained attending psychiatrists according to the *Diagnostic and Statistical Manual of Mental Disorders, Fifth Edition* (17). We included patients with MDD who visited the outpatient clinic of the Kyorin University Hospital and Sodegaura Satsukidai Hospital from April 2019 to March 2020. Patients were included if they were in clinically remission for at least 8 weeks prior to the investigation. Patients were excluded for the following criteria: (1) comorbid severe physical diseases, (2) dementia, (3) alcohol or substance abuse, (4) shift workers, (5) suicidal ideation, and (6) other sleep disorders, including sleep apnea syndrome, restless legs syndrome, periodic limb movement disorder, and narcolepsy and related disorders. As a result, 33 patients with MDD were included in this study.

### Assessments

Objective sleep was assessed using actigraphy (Actiwatch Spectrum, Philips Respironics). All participants were instructed to wear the watch for 14 consecutive days and remove the device only for water-based activities. We used sleep parameters derived from actigraphy software including bed in time, sleep latency, total sleep time, sleep efficiency, and wake-up time. The midpoint of sleep was calculated with the average time point between bed in time and wake-up time. Subjective sleep disturbances was assessed by the Insomnia Severity Index (ISI), which is a 7-item brief screening measure of insomnia and as an outcome measure in treatment research (18). We also evaluated the diagnosis of circadian rhythm sleep-wake disorders based on the International Classification of Sleep Disorders-third edition (ICSD-3) (19).

Cognitive function was assessed using the Japanese version of the Brief Assessment of Cognitive in Schizophrenia (BACS-J) (20). The BACS-J indicates an individual’s neurocognitive function in several major domains, including verbal memory, working memory, executive function, attention and processing speed, motor speed, and verbal fluency. It also provides a composite score of all the domains. We performed the BACS-J between 10 AM and 1 PM depending on the patients’ visiting time at the hospital.

Social functional impairment was assessed using the World Health Organization Disability Assessment Schedule (WHO-DAS), a self-administered questionnaire. Items were assessed on a 5-point scale (1 = none to 5 = extreme or cannot do), with a higher subscale score indicating social functional impairment. Quality of life (QOL) was assessed using the Euro QOL 5 dimensions (EQ5D) (21), a self-administered questionnaire. Items were assessed on a 3-point scale (1 = no problems to 3 = extreme problems).

We also evaluated patients’ demographics and characteristics [i.e., age, sex, education level (college graduate), employment status, marital status, living alone, family history of psychiatric disorders, onset age of MDD, and medication identified at the baseline].

### Statistical analysis

The Pearson correlation coefficient was used to compare the actigraphic parameters and BACS-J, WHO-DAS, and EQ5D scores. The Mann-Whitney U-test was used to compare BACS-J, WHO-DAS, and EQ5D scores between patients who took psychotropic medications (selective serotonin reuptake inhibitors, serotonin noradrenaline reuptake inhibitors, tricyclic antidepressants, noradrenergic and specific serotonergic antidepressants, antipsychotics, lithium, valproate, lamotrigine, benzodiazepines, ramelteon, and suvorexant) and those who did not. All statistical analyses were performed using SPSS, version 28 for Windows (IBM Corp., Armonk, NY, USA). A *p*-value <0.05 was considered to indicate a statistically significant difference.

## Results

**FIGURE 1:**
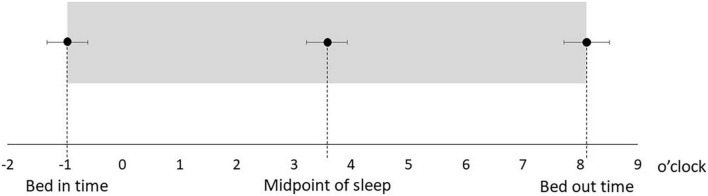
Actual sleep-wake schedule.

**FIGURE 2:**
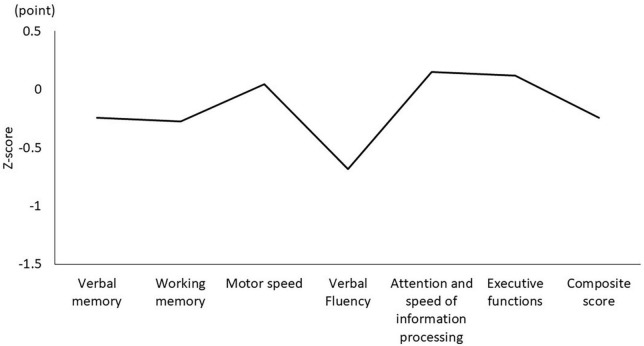
Composite score and each domain of the BACS-J. BACS-J: Japanese version of the Brief Assessment of Cognition in Schizophrenia.

Table 1 shows the characteristics of the study participants. The average age was 50.5 ± 14.3 years, and 17 (51.5%) were male (Table 1). Figure 1 shows the actual sleep schedule. The average time in bed was –0.96 ± 1.9 o’clock, sleep latency was 20.4 ± 12.4 min, total sleep time was 512 ± 89.9 min, arousal time was 56.7 ± 25.7 min, sleep efficiency was 83.6 ± 6.4%, wake time was 7.9 ± 2.0 o’clock, bed out time was 8.1 ± 2.1 o’clock, and the midpoint of sleep was 3.7 ± 1.8 o’clock (Figure 1). Of 33 MDD patients, six (18.2%) met the diagnostic criteria for DSWPD according to ICSD-3 criteria. There was no significant difference in the BACS-J, WHO-DAS, and EQ5D scores between patients who took psychotropic medications (selective serotonin reuptake inhibitors, serotonin noradrenaline reuptake inhibitors, tricyclic antidepressants, noradrenergic and specific serotonergic antidepressants, antipsychotics, lithium, valproate, lamotrigine, benzodiazepines, ramelteon, and suvorexant) and those who did not. Figure 2 shows the z-scores of the BACS-J. The average score of verbal memory was –0.24 ± 1.00, that of working memory was –0.27 ± 1.06, that of motor speed was 0.04 ± 0.84, that of verbal fluency was –0.68 ± 1.23, that of attention and speed information processing was 0.15 ± 1.43, that of executive functions was 0.12 ± 1.44, and the composite score was –0.24 ± 1.22 (Figure 2).

**TABLE 1:** Participant characteristics.

	Patients with major depressive disorder (*N* = 33) Mean ± SD or number (%)
Age (years)	50.5 ± 14.3
Sex (male/female)	17/16 (51.5)
College graduate (yes/no)	27/6 (81.8)
Employed (yes/no)	21/12 (63.6)
Married (yes/no)	12/21 (36.4)
Living alone (yes/no)	9/24 (27.3)
Family history of psychiatric disorders (yes/no)	12/21 (36.4)
Onset age of MDD (years)	40.0 ± 13.3
ISI total score (point)	10.24 ± 5.18
WHO-DAS total score (point)	31.38 ± 21.92
EQ-5D total score (point)	6.55 ± 1.44
**Medications identified at baseline**	
SSRI (yes/no)	9/24 (27.3)
SNRI (yes/no)	8/25 (24.2)
Tricyclic antidepressants (yes/no)	5/28 (15.2)
NaSSA (mirtazapine) (yes/no)	9/24 (27.3)
Antipsychotics (yes/no)	10/23 (30.3)
Lithium (yes/no)	2/31 (6.06)
Valproate (yes/no)	1/32 (3.03)
Lamotrigine (yes/no)	2/31 (6.06)
Benzodiazepines (yes/no)	17/16 (51.5)
Ramelteon (yes/no)	4/29 (12.1)
Suvorexant (yes/no)	5/28 (15.2)

MDD, major depressive disorder; SD, standard deviation; ISI, Insomnia Severity Index; WHO-DAS, World Health Organization Disability Assessment Schedule; EQ5D, Euro QOL 5 dimensions; SSRI, selective serotonin reuptake inhibitor; SNRI, serotonin noradrenaline reuptake inhibitor; NaSSA, noradrenergic and specific serotonergic antidepressant.

**FIGURE 3:**
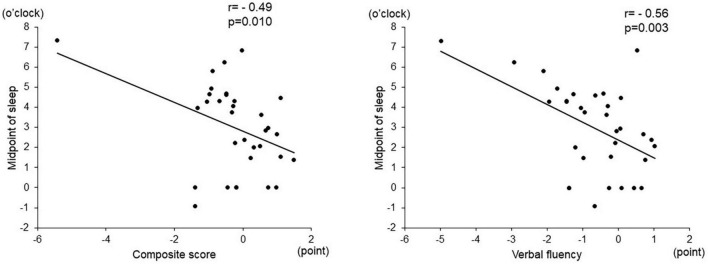
Correlation between the midpoint of sleep and BACS-J scores. The midpoint of sleep shows a significant negative correlation with verbal fluency (*r* = –0.56, *p* = 0.003) and the composite score (*r* = –0.49, *p* = 0.010). BACS-J | Japanese version of the Brief Assessment of Cognition in Schizophrenia.

**FIGURE 4:**
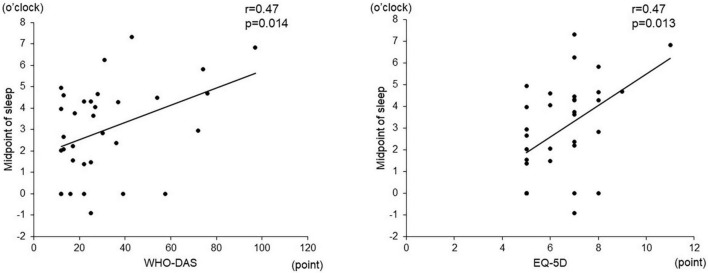
Correlation between the midpoint of sleep and WHO-DAS and EQD scores. The midpoint of sleep shows a significant correlation with the WHO-DAS (*r* = 0.47, *p* = 0.014) and EQ5D scores (*r* = 0.47, *p* = 0.013). WHO-DAS: World Health Organization Disability Assessment Schedule, EQ5D: Euro QOL 5 dimensions.

Among the objective sleep parameters in the actigraphic assessment, only the midpoint of sleep showed a significant negative correlation with verbal fluency (*r* = –0.56, *p* = 0.003) and the composite score (*r* = –0.49, *p* = 0.010). There was no significant correlation between other actigraphic sleep parameters (sleep latency, total sleep time, and sleep efficiency) and z-scores of the BACS-J (Table 2 and Figure 3). There was also no significant relationship between the subjective sleep quality in ISI and BACS-J scores. Similarly, among the objective sleep parameters in the actigraphic assessment, only the midpoint of sleep showed a significant correlation with the WHO-DAS (*r* = 0.47, *p* = 0.014) and EQ5D scores (*r* = 0.47, *p* = 0.013; Figure 4). There was no other significant correlation between the sleep parameters (sleep latency, total sleep time, and sleep efficiency) and WHO-DAS and EQ5D scores. There was also a significant relationship between the subjective sleep quality in ISI score and WHO-DAS and EQ5D scores.

**TABLE 2:** Correlation between sleep parameters and BACS-J scores.

	Verbal memory	Working memory	Motor speed	Verbal fluency	Attention and speed of information processing	Executive functions	Composite score
Sleep latency	*r* = –0.14	*r* = –0.07	*r* = –0.10	*r* = –0.03	*r* = 0.03	*r* = –0.30	*r* = –0.16
	*p* = 0.471	*p* = 0.721	*p* = 0.607	*p* = 0.880	*p* = 0.883	*p* = 0.120	*p* = 0.404
Total sleep time	*r* = –0.02	*r* = 0.01	*r* = –0.13	*r* = 0.05	*r* = –0.06	*r* = 0.03	*r* = –0.03
	*p* = 0.909	*p* = 0.956	*p* = 0.516	*p* = 0.794	*p* = 0.741	*p* = 0.897	*p* = 0.897
Sleep efficiency	*r* = –0.05	*r* = –0.09	*r* = 0.03	*r* = 0.01	*r* = 0.00	*r* = 0.09	*r* = 0.01
	*p* = 0.816	*p* = 0.648	*p* = 0.896	*p* = 0.958	*p* = 0.995	*p* = 0.657	*p* = 0.951
Midpoint of sleep	*r* = –0.26	*r* = –0.23	*r* = 0.00	*r* = –0.56	*r* = –0.36	*r* = –0.37	*r* = –0.49
	*p* = 0.198	*p* = 0.245	*p* = 0.999	*p* = 0.003	*p* = 0.065	*p* = 0.061	*p* = 0.010

BACS-J, Japanese version of the Brief Assessment of Cognition in Schizophrenia.

## Discussion

Previous studies have reported a significant association between delayed sleep-wake phase disorder and depression. Several studies suggested that depressive symptoms were common in patients with delayed sleep-wake phase disorder (22, 23). Circadian rhythm misalignment could increase vulnerability to developing depressive symptoms. Few studies have investigated the prevalence of delayed sleep-wake phase disorder in patients with MDD. Although the prevalence of delayed sleep-wake phase disorder was higher in bipolar disorder than in MDD, approximately 10% of patients with MDD had delayed sleep-wake phase disorder (24).

Although the exact mechanisms linking delayed sleep-wake rhythm and mood disturbances are not yet fully understood, there is a complex and bidirectional relationship between delayed sleep-wake rhythm and the pathophysiology of MDD. A delayed sleep-wake rhythm may cause depressive symptoms in some patients, while residual depressive symptoms may cause delayed sleep-wake rhythm in others. A previous study reported that circadian misalignment in patients with delayed sleep phase disorder is strongly associated with depressive symptoms even after controlling for other confounding factors (25), which implies that delayed sleep-wake rhythm could worsen depressive symptoms and possibly cause the development of MDD pathophysiology. Of note, a preliminary randomized-controlled trial suggested that treatment of delayed sleep phase syndrome with melatonin significantly improved depressive symptoms (26), which implies that delayed sleep-wake rhythm could trigger worsening depressive symptoms and could be a treatment target in MDD patients comorbid with delayed sleep-wake rhythm. Another possible relationship between delayed sleep-wake rhythm and mood dysfunction is that cognitive dysfunction in patients with MDD might make social adaptation worse, leading to weak social cues and then a sleep-wake phase delay. A previous study suggested that cognitive dysfunctions were a principal mediator of social impairment in patients with MDD (10), which might consequently cause a delayed sleep-wake phase. Further longitudinal studies will be needed to confirm the relationship between delayed sleep-wake rhythm and the pathophysiology of MDD.

A previous systematic review and meta-analysis suggested that cognitive dysfunctions across the domains of attention, executive function, and memory were core features of cognitive dysfunction in patients with MDD compared to healthy controls (27). A previous study that used BACS as the same cognitive assessment as ours reported that verbal and working memory, attention and processing speed, and the composite score were significant impaired domains in later life in patients with MDD (9). Another study that investigated the relationship between BACS scores and functional capacity reported that BACS composite scores were moderate to strongly associated with functional capacity in patients with MDD. However, the present study’s results suggested that verbal fluency and the composite score of the BACS-J were impaired in our patients with MDD. Although the reason for this inconsistency among the studies was unclear, the composite score of the BACS-J might be representative of cognitive dysfunction in patients with MDD. Because few studies have shown a relationship between a delayed sleep-wake phase and cognitive dysfunction (7), it is difficult to interpret this result. One possible explanation for this relationship was that the delayed sleep-wake phase, which usually caused physical and mental dysfunction, daytime sleepiness, and loss of concentration, might deteriorate daytime cognitive function. Further studies are needed to confirm the relationship.

Few studies have investigated the relationship between sleep disturbances, social dysfunction, and quality of life in MDD patients. A previous observational study suggested that MDD patients with subjective sleep disturbances had more severe social dysfunction than MDD patients without sleep disturbances (28). Another study evaluated the relationship between subjective sleep problems and functional impairment in depressed college students and reported that depressed students with subjective sleep disturbances had a more severe functional impairment, including QOL, physical dysfunction, and cognitive dysfunction, than students without sleep disturbances (29). These findings suggested that subjective sleep disturbances might negatively affect functional impairment in patients with MDD. The result of our study was in line with these findings, which showed a significant association between subjective sleep quality and QOL and social function.

Conversely, our study only suggested an association between the midpoint of sleep and functional impairment in the objective actigraphic assessment. Because no other study investigated the association between functional impairment and subject/objective sleep problems simultaneously, it is difficult to understand this discrepancy. Previous forced desynchrony studies in the laboratory might support our result that delayed sleep-wake rhythm could worsen functional impairments, including sleepiness and cognitive dysfunction (30, 31). These experimental studies also suggested circadian misalignment could cause functional impairment in MDD patients and healthy individuals. Further studies are needed to clarify whether the impact of circadian misalignment on functional impairment is different between MDD patients and healthy individuals.

This study has several limitations. First, the sample size of this study was small, which caused limitation of statistical power. Second, because this study had a cross-sectional design, it is difficult to draw any conclusion about the causal relationships between sleep disturbances and cognitive dysfunctions. Third, because we did not use the controls, it is difficult to interpret the result of this study. Fourth, because many patients refused to participate in this study, there could be selection bias in our study sample, which might affect the results of this study. For example, the average total sleep time might be longer, and wake up time might be later in our study patients than in typical MDD patients and normal controls. Fifth, we performed the BACS-J between 10 AM and 1 PM depending on the patients’ visiting schedule at the hospital, which might affect scores of BACS-J, especially in patients with delayed sleep-wake rhythm. Sixth, although we evaluated the influences of psychotropic medications on daytime functions, we could not detect a significant statistical association between psychotropic medications and daytime functions, possibly due to the small sample size or that the psychotropic medications affected daytime functions. Seventh, although we used actigraphy as an objective sleep parameter, a polysomnographic assessment would be desirable to confirm the relationship between sleep disturbances and functional impairment in MDD patients.

## Conclusion

In conclusion, this study’s results suggested that the delayed sleep-wake phase is associated with cognitive dysfunction, social dysfunction, and deteriorated QOL in patients with MDD. To prevent relapse of depressive symptoms, clinicians should pay attention to the disturbance of sleep-wake rhythm in patients with MDD in clinical settings.

## Data availability statement

The original contributions presented in this study are included in the article/supplementary material, further inquiries can be directed to the corresponding author.

## Ethics statement

The studies involving human participants were reviewed and approved by the Ethics Committee of Kyorin University. The patients/participants provided their written informed consent to participate in this study.

## Author contributions

YT: drafting the manuscript, organizing and implementation of this study, and interpreting the data. YT, YK, KW, and TH: recruiting the participants. MI: collecting the data. YT, MI, and YN: statistical analysis. All authors took part in revising the manuscript and approved the final version of the manuscript.
